# Comparison and evaluation of the morphology of crowns generated by biogeneric design technique with CEREC chairside system

**DOI:** 10.1371/journal.pone.0227050

**Published:** 2020-01-16

**Authors:** Fang Wang, Qingqing Tang, Shuang Xi, Ruirui Liu, Lin Niu

**Affiliations:** 1 Key Laboratory of Shaanxi Province for Craniofacial Precision Medicine Research, College of Stomatology, Xi’an Jiaotong University, China; 2 Clinical Research Center of Shaanxi Province for Dental and Maxillofacial Diseases, College of Stomatology, Xi’an Jiaotong University, China; Virginia Commonwealth University, UNITED STATES

## Abstract

**Objectives:**

To better guide clinicians to choose the appropriate chairside system, we compared and evaluated the morphology of crowns generated by three different biogeneric design modes (biogeneric copy (BC), biogeneric individual (BI), and biogeneric reference (BR)) of the CEREC software.

**Methods:**

Maxillary and mandibular casts were obtained from twelve volunteers and digital impressions were acquired. All ceramic crown preparations of all right maxillary central incisors were prepared and digital impressions were taken. Then, crowns were automatically designed under BC, BI and BR modes separately and their morphologies were evaluated by six doctors. The “optimal fitting alignment” and “3D analysis” functions of the Geomagic Qualify software were carried out between original teeth and auto-generated full crowns. The auto-generated crowns were modified by a technician according to clinical criteria and the adjustment time was recorded. The discrepancies between technician modified crowns and the auto-generated full crowns were evaluated with the same functions in the Geomagic Qualify software.

**Results:**

The subjective evaluation results of BC group were significantly better than those of BI and BR group (p < 0.05). Compared with the original teeth and modified crowns, auto-generated crowns in BC group all had the smallest differences, followed by BR and BI group (p < 0.05). BC group needed the shortest adjustment time than BI and BR group (p < 0.05).

**Conclusions:**

Using crowns generated by BC mode is more aesthetic and suitable in clinics use than those generated by BI and BR modes and can reduce clinic adjustment time.

## Introduction

Since application of computer-aided design and computer-aided manufacturing (CAD/CAM) technology in the late 1980s, the restorative dentistry has been changing from manual fabrication of tooth restoration towards a more computerized fabrication[1,2]. The advanced CAD/CAM technology can be divided into three systems according to their production methods. In the chairside system, the dentist scans the prepared tooth digitally, makes chairside restoration, and seats it at one visit. In the laboratory system, the laboratory technician scans the model made from impression and uses CAD/CAM to generate restoration. In the centralized production system, the dentist captures digital impression on chairside and sends data via Internet to the laboratory, where the technician uses CAD/CAM to generate restoration[3].

The chairside system is a viable alternative to traditional procedures with several advantages[3–6]. First, indirect repair can be completed in one visit, which eliminates the need for temporary restorations, increases the durability of dental tissue adhesion, and reduces postoperative sensitivity, thus improving the efficiency[5,7]. Second, the optical image of the prepared tooth can be obtained directly with an intraoral scanner without the traditional impression procedure, which can improve the patient's comfort[3,8,9]. Third, it uses new and almost defect-free industrial prefabrication and control materials[10]. Fourth, it has better quality and reproducibility compared with the traditional process[11]. Fifth, data storage is commensurate with the standardized production chain[10].

The digital design software of chairside CAD/CAM system has obvious technical characteristics oriented to the needs of clinicians. 1) The software design function is specifically suitable for the needs of chairside routine treatment, but does not pursue comprehensiveness. 2) The embedded design process and step-by-step instructions make it less flexibility but more convenience for doctors to learn and use. 3) The empirical parameters for prosthesis design are preset in the software. Thus, the users do not need to frequently adjust the parameters during the design, making the design more fluent and efficient, and eventually saving chairside time. 4) The software uses preset parameters and intelligent algorithms to reduce the difficulty for complex links design and minimize the chairside operation time[12]. Considering that most clinicians are not systematically trained for crown design, the characteristics of chairside system can help clinicians to accomplish better prosthetics treatment and shorten the clinic operation time.

The generation of morphology of CAD/CAM restorations using CAD software is mainly based on the standard libraries[1,13]. However, the standard automatic adaptation process is difficult for individual clinical defects. Thus, clinicians have to adopt manual design tools, making the design time-consuming and possibly affecting the strength of restorations[14–20].

CEREC software is a major application of CAD/CAM technology in dental reconstruction[3,11]. The system adopts an intelligent algorithm named biogeneric design mode for morphological restoration. Its scientific basis is the existence of morphological relationship between teeth, which can be expressed by mathematical functions[16,17,21]. Based on the mathematical descriptions, using the algorithm of biogeneric intelligent restoration morphology design of CEREC software, one can obtain information from three-dimensional (3D) database of the adjacent and opposite teeth, pre-prepare tooth and homonym tooth, and automatically generate crowns with morphology suitable for patient’s personalized dentition and occlusal characteristics. Compared with the traditional standard methods, it can greatly reduce the workload and time of manual adjustment[2,13,16,18,22].

Biogeneric design includes three modes: biogeneric copy (BC), biogeneric individual (BI), and biogeneric reference (BR). In the BC mode, the anatomical structure of the teeth is replicated before preparation, and the residual teeth are modified with the help of the bio-reconstructed scheme so as to keep the morphology and functional unchanged. In the BI mode, the preparations are analyzed based on the 3D database included hundreds of caries-free and complete crown surface scans in the software, and the remaining teeth are used to bio-reconstruct the missing teeth based on the database and algorithm. In the BR mode, after determining which tooth to use as the reference for calculating the prosthesis scheme, the design of the restoration derived from the reference tooth is made to achieve the desired morphology [5,11,18,23].

There have been some studies about the biogeneric design techniques [1,11,13,16,18,22,24], which were focused on the comparison of occlusion surface rather than on the overall morphology of the crown. What’s more, these studies used the 2D method to compare morphological differences, which is less objective than the results of 3D comparison.

The 3D differences between two datasets can be analyzed by superimposing appropriate detection software. In most cases, these software programs use the “best fit algorithm” and “3D analysis” to compare 3D datasets[24]. In order to describe the accuracy of digital 3D model, the “trueness” and “precision” parameters are adopted. Referring to ISO Norm 5725–1, “trueness” refers to the consistency between the arithmetic mean of a large number of test results and the true or acceptable reference values. The term “precision” refers to the consistency of test results, usually expressed in standard deviations. Geomagic qualify 12.0 (Geomagic; Morrisville, USA) is a reverse checking software, which can use “the best fit algorithm” and “3D analysis” to quickly detect the differences between 3D datasets[4,25].

The purpose of this study was to compare and evaluate the differences of crown morphology generated BI, BC and BR modes of the CEREC software with the hope to better guide clinicians to choose the appropriate chairside design strategy.

## Materials and methods

### Optimal impression taking and tooth preparation

**Fig 1:**
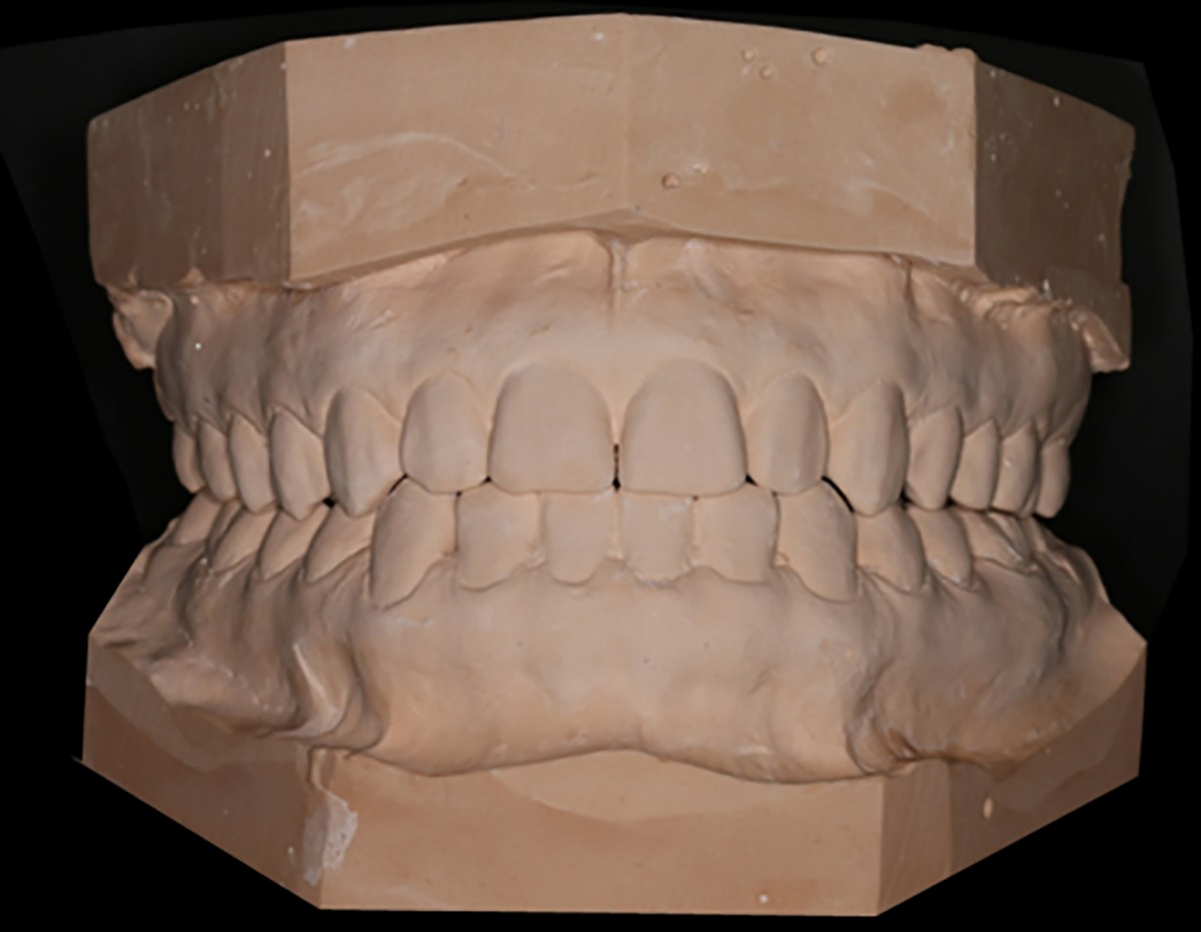
Gypsum model with uniform perfusion.

Twelve volunteers with intact, natural and symmetrical maxillary central incisors were selected for the study. All participants agreed to participate in the study and have signed the informed consents. The study has been approved by Medical Ethics Committee of Stomatology Hospital of Xi'an Jiaotong University (xjkqll[2016]035). After maxillary and mandible silicone rubber impressions (Honigum blue, DMG, Hamburg, Germany) were taken, plaster replicas were made with type IV gypsum (Die-Stone, Heraeus Kulzer, USA) from the silicone rubber impressions (Fig 1). The dentitions and the centric bite registrations were scanned using an opto-electronic intraoral scanner (CEREC-3D, Sirona, Bensheim, Germany) according to the manufacturer’s instructions and the original model datasets were obtained.

**Fig 2:**
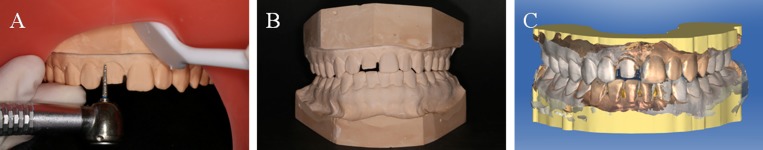
Preparing the right maxillary central incisor (11) (A), the Gypsum model of the preparation (B), the virtual image of the preparation (C).

Full crown of all right maxillary central incisors of the twelve volunteers were prepared by the same experienced clinician (Fig 2) and the virtual images of the preparations and the centric bite registrations were collected according to the same protocol.

### Automatic generating of crowns

**Fig 3:**
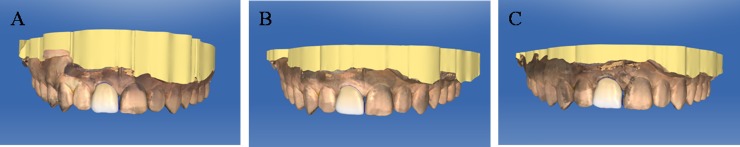
The pictures of the auto-generated crowns by the biogeneric function. (A. BC mode, B. BI mode, C. BR mode).

After the virtual models were trimmed and the preparation margins were placed, the insertion axes were determined to be parallel to the axis of the respective tooth and perpendicular to the occlusal plane. Then the prepared teeth were reconstructed via the biogeneric function (BC, BI and BR modes) of the CEREC software (Sirona Dental Systems GmbH, Bensheim, Germany). In total, 36 full crowns were automatically generated, among which, 12 were made by the BC mode, 12 by the BI mode and 12 by the BR mode (Fig 3).

### Subjective evaluation of auto-generated crowns

The morphology of all auto-generated crowns from three different modes was subjectively assessed in a double-blind manner by six doctors who did not participate in the experiment and assigned to different scores based on their similarity to the homonym teeth. The score 3 indicates that the crown has excellent quality, showing high aesthetics and high similarity with homonym tooth; score 2 means the crown has medium quality, showing moderate aesthetics and moderate similarity with homonym tooth; and score 1 indicates the crown has poor quality, showing poor aesthetics and poor similarity with homonym tooth.

### Evaluation of the discrepancy between the auto-generated crowns and the original teeth

**Fig 4:**
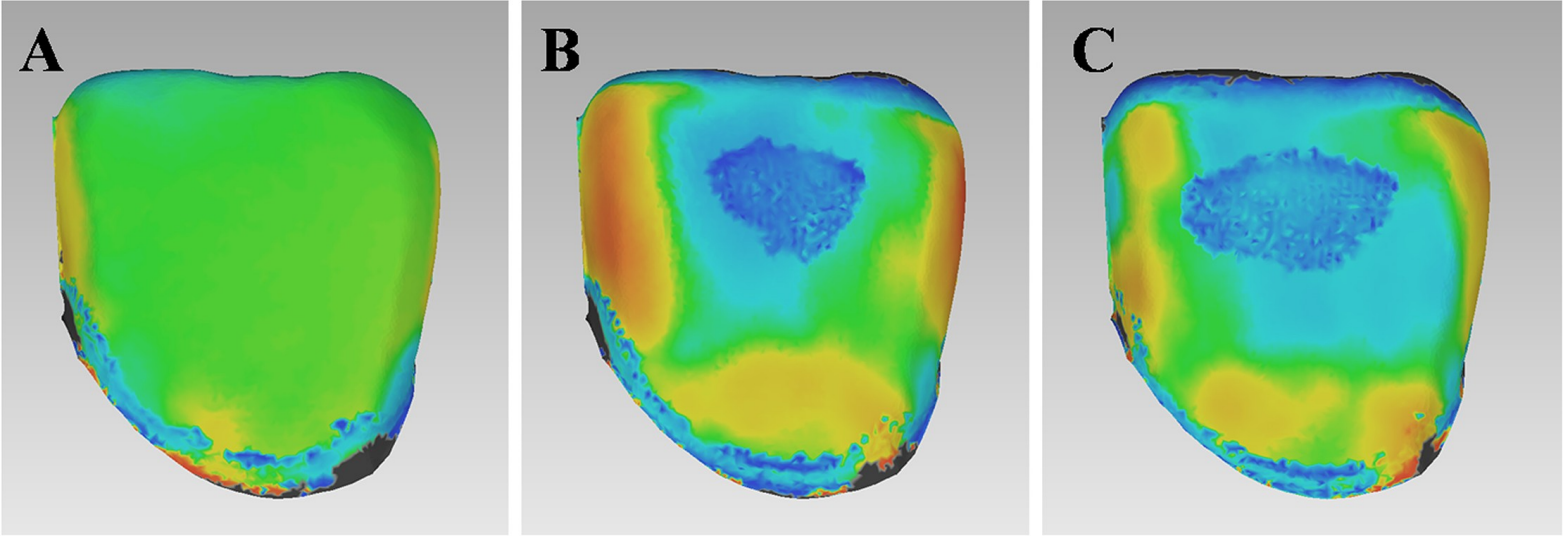
3D color maps for comparing the auto-generated crown and original tooth using “optimal fitting alignment” and “3D analysis”. (A. BC mode, B. BI mode, C. BR mode).

The datasets of the original teeth and the auto-generated full crowns were transformed into STL format. The datasets of each patient’s original crowns (including BC, BI and BR) were set as the reference group, and the datasets of the auto-generated crowns (including BC, BI and BR) were set as experimental groups. The differences of datasets in reference and experimental groups were compared using the Geomagic qualification software with the functions of “the optimum fit algorithm” and “3D analysis”. The average positive and negative deviations, standard deviations and the root mean square (RMS) values were obtained from the deviations output of Euclidean distance between the datasets of auto-generated crowns and original dental datasets (Fig 4).

### Evaluation of the adjustment time and the discrepancy between the auto-generated crowns and the modified crowns

A total of 36 auto-generated crowns were modified by an experienced technician according to the available clinical standards and the adjustment time of each crown was recorded. The datasets of the modified crowns were obtained and transformed into STL format. The datasets of each patient’s modified crown (including BC, BI and BR) were set as reference groups, and the datasets of the auto-generated crowns (including BC, BI and BR) were set as experimental groups. The differences of datasets in reference and experimental groups were compared and analyzed as mentioned in Section 2.4.

### Statistical analysis

All data were subjected to statistical analysis using SPSS statistical software (version 25.0). The adjustment time and the subjective evaluation results were analyzed via the nonparametric Kruskal-Wallis test (α level 0.05).All differences in the average positive and negative deviations, standard deviations and RMS values between the auto-generated crowns and the original teeth, as well as between the auto-generated crowns and the modified crowns were analyzed by one-way ANOVA with least significant difference (LSD) post hoc tests (α level for all tests 0.05). The heterogeneity of variances between two groups was measured by the Levene’s test (p<0.05). A value of p<0.05 was considered significantly different.

## Results

**Fig 5:**
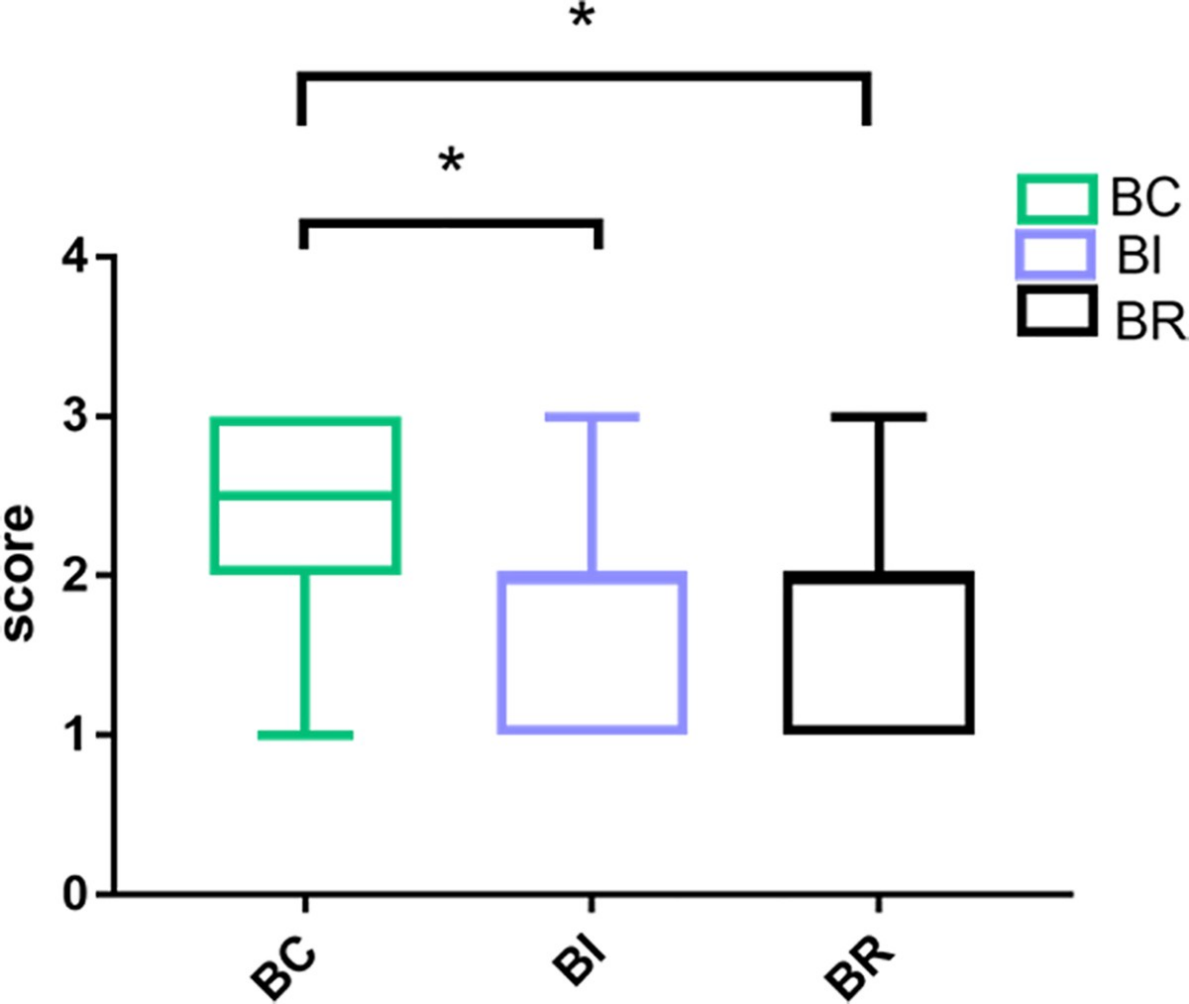
The subjective evaluation results of BC, BI and BR modes. * p <0.05.

The subjective evaluation score was 2.38 for BC, 1.80 for BI and 1.83 for BR, respectively. Furthermore, the Kruskal-Wallis test showed significant differences among the three groups (p<0.05). The comparisons between two groups showed significant difference between BC and BI groups as well as between BC and BR groups (p<0.05), but not between BI and BR groups (p>0.05). Overall, the subjective evaluation results of BC group were significantly better than those of BI and BR group (Fig 5).

**Fig 6:**
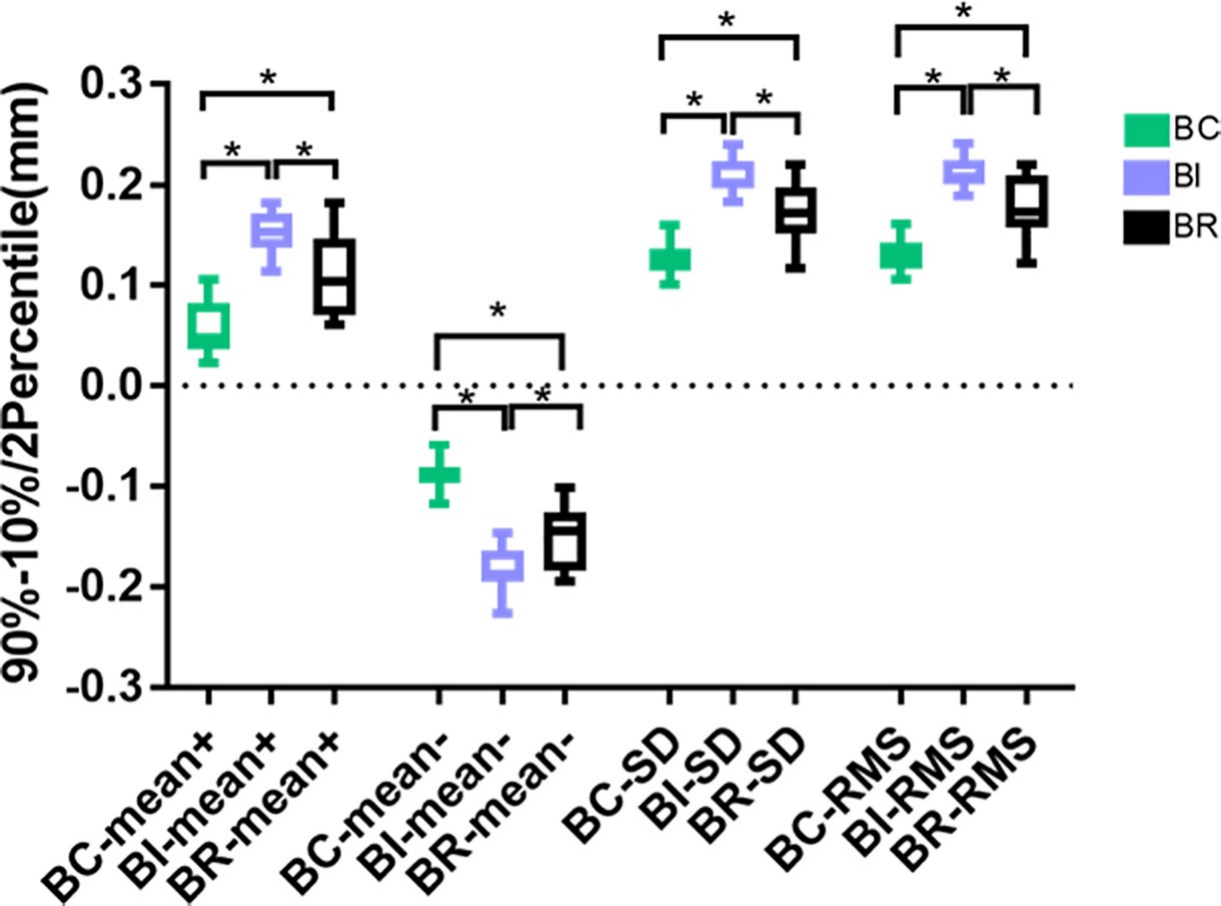
The average positive and negative deviations, standard deviations and RMS values between the auto-generated crown groups and the original tooth groups. * p <0.05.

Between the auto-generated crown groups and the original tooth groups, the average positive deviations were 0.058 mm for BC, 0.151 mm for BI, and 0.111 mm for BR; the negative deviations were -0. 089 mm for BC, -0. 183 mm for BI, and -0. 151 for BR; the standard deviations were 0.127 for BC, 0.208 for BI, and 0.171 for BR; and the RMS values were 0.131 for BC, 0.212 for BI, and 0.178 for BR. Levene’s test of variance heterogeneity was not significant between the groups (p>0.05). One-way ANOVA analysis showed significant differences between the original teeth and the auto-generated crowns in BC, BI and BR modes (p<0.05). LSD post hoc tests showed that all discrepancies were statistically significant. Compared with the original tooth morphology, BC group had the smallest difference, followed by BR group and BI group (Fig 6).

**Fig 7:**
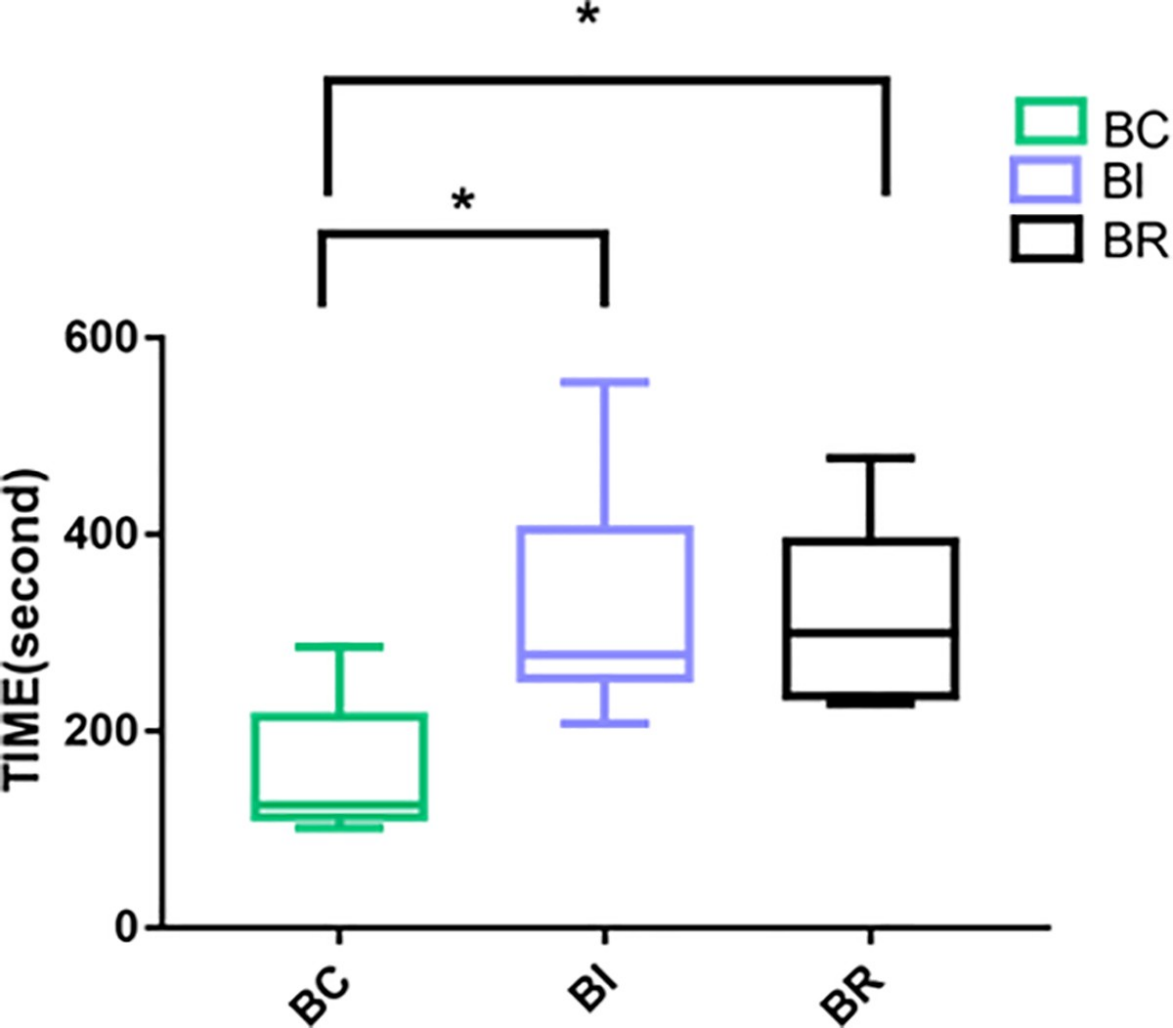
The average adjustment time of BC, BI and BR modes. * p < 0.05.

The average adjustment time was 160 s for BC, 321 s for BI, and 320 s for BR. The Kruskal-Wallis test showed significant differences among the three groups (p < 0.05). The comparisons between two groups showed significant difference between BC and BI groups as well as between BC and BR groups (p < 0.05), but not between BI and BR groups (p > 0.05).BC group needed the shortest adjustment time (Fig 7).

**Fig 8:**
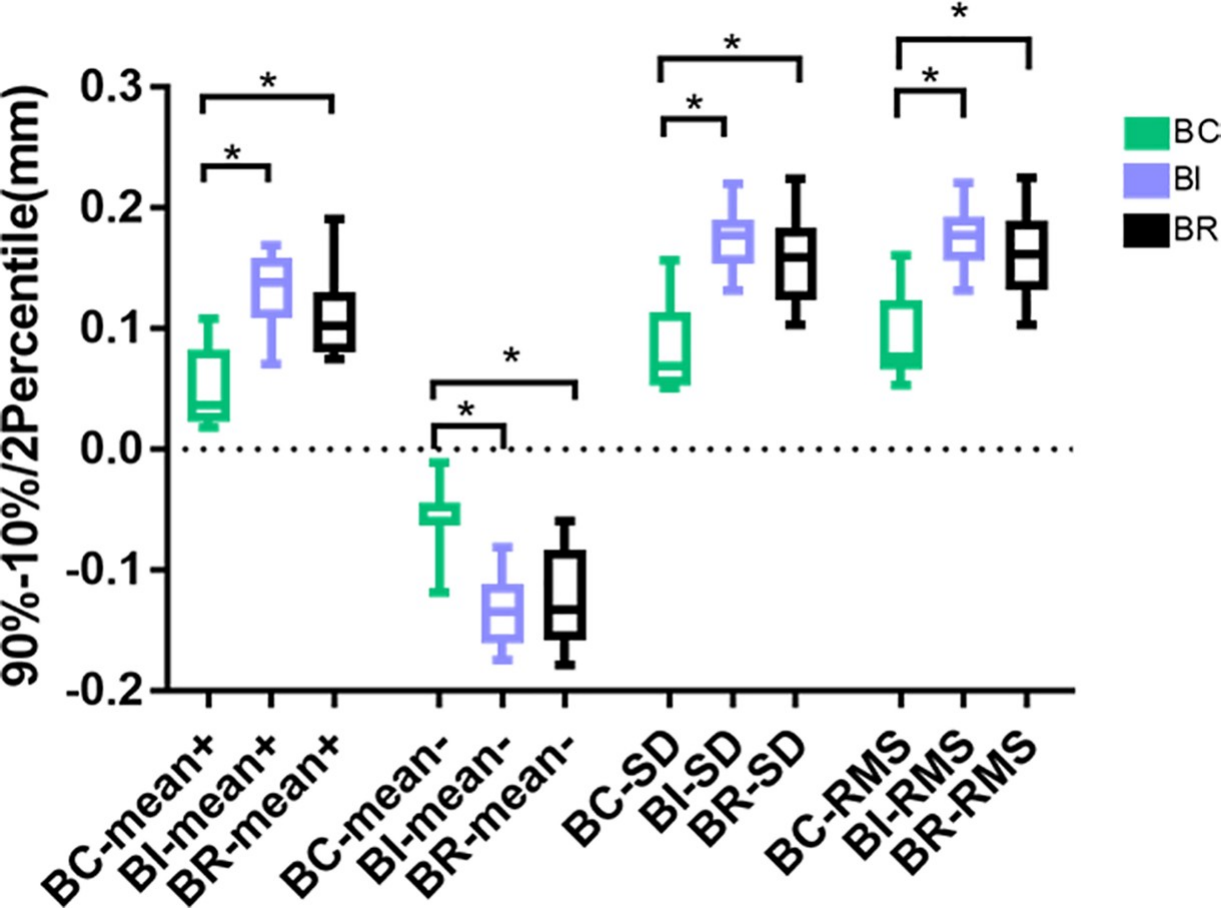
The average positive and negative deviations, standard deviations and RMS values between the auto-generated crowns and the modified crowns. * p <0.05.

Between the auto-generated crowns and the modified crowns, the average positive deviations were 0.050 mm for BC, 0.132 mm for BI, and 0.109 mm for BR; the negative deviations were -0.062 mm for BC, -0.132 mm for BI, and -0.124 for BR, the standard deviations were 0.085 for BC, 0.174 for BI, and 0.157 for BR; and the RMS values were 0.093 for BC, 0.176 for BI, and 0.164 for BR. Levene’s test of variance heterogeneity was not significant between the groups (p>0.05). One-way ANOVA analysis showed significant differences between the auto-generated crowns and the modified crowns in BC, BI and BR modes (p<0.05). LSD post hoc tests showed significant discrepancies in the mean positive and negative deviations, standard deviation and RMS value between BC and BI as well as between BC and BR groups (p < 0.05). There was no significant difference between BI and BR(p > 0.05). Before and after technician’s adjustment, BC group had the smallest differences compared with the BR and BI groups, and BR and BI groups had no significant difference (Fig 8).

## Discussion

The natural and harmonious morphology is of great significance to a crown[17,18]. Reconstructing functional occlusal and axial surfaces in coordination with adjacent teeth and opposite teeth is very important for the stability of the stomatognathic system[19,22,26]. Accurately reconstructing morphology can reduce the adjustment time, thereby reducing the chairside operation time. In addition, less grinding means that the crown can be better positioned without damaging the material[27].

In clinic, the design of crown using the chairside system is mainly completed by clinicians independently. However, most clinicians lack design knowledge about crown morphology compared with technicians[3,5]. Therefore, it is very important for clinical application of chairside system to automatically generate crowns suitable for patients’ individual dentitions and occlusal characteristics. Using the new “biogeneric design mode” developed by CEREC software, one can automatically generate crowns for individuals. This method includes three modes, namely BC, BI and BR, each of which can make up for the clinicians weakness to a certain extent and improve the prosthetic effect of patients.

In this study, we selected patients with intact, natural and symmetrical maxillary central incisors. On this premise, the “biogeneric design mode” (BC、BI and BR) of CERCE software was used to design crowns. The results show that the crown morphologies generated by BC mode were more similar to the original teeth than those generated by BR and BI modes. The subjective evaluation results of BC, BI and BR modes further confirmed that the morphology of crowns generated by BC mode were superior to those by BI and BR modes.

The teeth morphologies will change due to long-term erosion, attrition and abrasion. Reconstruction of the stomatognathic systems is a long and complex process[28]. Sudden changes in teeth morphologies may cause disorder of the stomatognathic systems. For example, changes in protrusion of the adjacent faces may cause food impaction, changes in protrusion of the buccal and lingual sides may affect the balances of soft tissues and result in biting cheek or tongue, and changes in occlusal surfaces may cause occlusal interferences, abnormal vertical dimensions, and even temporomandibular joint disturbance syndrome[29]. If an intact tooth requiring root canal therapy, BC mode can be used to restore the original tooth morphology after treatment. For tooth that has been repaired by a comfortable and satisfactory temporary crown for a long time, BC mode can also be used to make formal restoration according to the morphology of the temporary crown[15,19,30].

For patients with removable dentures, if the remaining teeth need root canal therapy and crown restoration because of pulpitis, dental trauma or other diseases, the teeth morphology will change, which will make the original removable denture unable to completely seat. Therefore, ensuring morphology of the crowns consistent to that of the teeth before preparation can improve patients’ comforts, reduce the costs and shorten the treatment cycles[20]. If patients only have a few teeth left, but still remain good occlusal relationships, when one or several teeth need crown restorations, the original occlusal relationship will be lost after teeth preparation. In conventional prosthodontics, the occlusal relationship needs to be re-determined, which will prolong the treatment cycle and adversely affect the repairing effect. Thus, repairing tooth morphology with crowns similar to the original teeth will restore the original occlusal relationship and improve the treatment efficiency. For these two groups of patients, using BC mode to maintain the stability of crown morphology is of great significance to the health of the stomatognathic system.

However, in clinic, the teeth requiring crown restorations are mostly incomplete caused by caries, trauma and other reasons. If the morphology of its homonym tooth is intact, BR mode maybe is an effective design mode for generating a crown with morphology similar to the homonym tooth, especially for the anterior teeth[11,18]. This technique is helpful to achieve symmetry in anterior crown design[11,13]. The central incisors are the main feature of the esthetic smile, and should show a high degree of symmetry in the midline[19,23]. Traditional lab-made crown is difficult to achieve symmetry, and its success depends largely on the skills of dental technicians[23]. BR mode can very easily and quickly generate the line angle and incision edge morphology of homonym tooth[11,13]. For patients with aesthetic restoration of anterior teeth, the diagnostic wax-up can be duplicated to the final restorations to obtain satisfactory restoration results[1].

If the morphology of the original tooth and the homonym tooth are not good, BI mode can be used to design crowns. BI mode is based on mathematical algorithm and information of adjacent and opposite teeth to generate crown. So, it can generate crowns with more accurate adjacency and better occlusal relationships than BC and BR modes. Literatures have shown that BI mode can also obtain a good morphology, close to the original teeth[1,16,17,22]. Our result is opposite from the conclusion that crowns generated by BI mode had closer occlusal contacts to the original teeth than those by BC mode of a previous study evaluating the occlusal contact of crown generated by biogeneric design mode[18]. This discrepancy may be due to that the latter only involved occlusal surface and did not consider the axial morphology, while our study only included patients with complete and symmetrical central incisors.

In this study, we also compared the auto-generated crowns with the crowns adjusted by the technician, and calculated the adjustment time. It can be concluded that BC mode has the smallest difference and the shortest adjustment time, indicating that the morphology of the crown automatically generated by BC mode is more in line with the clinical requirements than those by BI and BR modes. Therefore, it can be inferred that in clinics, at the conditions specified in this experiment, the adjustment time and difficulty of crowns generated by BC mode are less than those by BI and BR modes. Therefore, using BC mode can reduce the operation time and operation difficulty for clinicians. Therefore, if patient’s original tooth morphology is intact and natural, BC mode can be a good chose for clinicians.

One requirement of chairside CAD/CAM system is the reasonable clinic adjusting time. Therefore, adjustment time of the prosthesis is one of the important indicators to evaluate the prosthesis[17,18]. In this study, the adjustment time was about 2–4 minutes. In another study using biogeneric design mode, the adjustment time was 4–5 minutes[13], which are largely less than the adjustment time required by other methods[31,32]. Designing prostheses with biogeneric design mode can significantly improve clinical efficiency.

In Geomagic qualification software, the “best fit alignment” and “3D analysis” functions are used to evaluate datasets by superposing them. This method is also used in some other studies to compare 3D datasets[7,33,34].With the “best fit alignment” and “3D analysis” functions, there will be positive and negative deviations between the experimental datasets and the reference datasets. These deviates are difficult to explain because the arithmetic mean of positive and negative deviations will leads to the result close to zero, which could not fully represent the actual divergence. In this study, positive deviation, negative deviation and standard deviation are used to estimate the difference between experimental datasets and reference datasets. According to these values, the average value of each group can be calculated. These average values are divided into positive and negative ranges. Calculating the average absolute values of Euclidean deviation for each group gives the average distance between the experimental datasets and the reference datasets for a single measurement point, regardless of whether it is located “above” or “below” the reference surface[4].

Our study has certain limitations. It only included patients with intact, natural and symmetrical maxillary central incisors, so the application range of the experimental results are limited. In the future, we will explore analyze teeth in different situations to determine the correct choice of BC, BI and BR modes for clinicians under other premises.

In order to better serve patients, it is necessary for clinicians to spend time on learning the knowledge of clinical available standards of crown morphology and the abilities of modifying crown’s morphology on the chairside design software. At the same time, relevant design software should be upgraded to optimize the system in order to automatically generate prostheses closer to clinical needs[2,5].

## Conclusion

Morphological comparison and evaluation of crowns generated by three kinds of biogeneric design modes (BI, BC and BR) of CEREC software indicated that for patients with intact, natural and symmetrical maxillary central incisors, the crowns generated automatically by BC mode 1) can restore the natural morphology more accurately than those by BI and BR modes and 2) are closer to the modified crowns than those by BI and BR modes in shorter adjustment time, further indicating that the morphology of the crowns automatically generated by BC modes are more suitable for use in clinics. Moreover, subjective evaluation of clinicians confirmed that the morphology of crowns generated by BC mode is more aesthetic than that of crowns generated by BI and BR modes.

## Supporting information

S1 Fig (TIF)

S2 Fig (TIF)

S3 Fig (TIF)

S4 Fig (TIF)

S5 Fig (TIF)

S6 Fig (TIF)

S7 Fig (TIF)

S8 Fig (TIF)

S9 Fig (TIF)

S10 Fig (TIF)

S11 Fig (TIF)

S12 Fig (TIF)

S13 Fig (TIF)
