# Supplementary figures and images for: Comparison and evaluation of the morphology of crowns generated by biogeneric design technique with CEREC chairside system
Source: PLoS One. 2020 Jan 16;15(1):e0227050. doi: 10.1371/journal.pone.0227050 (PMC6964887; doi:10.1371/journal.pone.0227050)

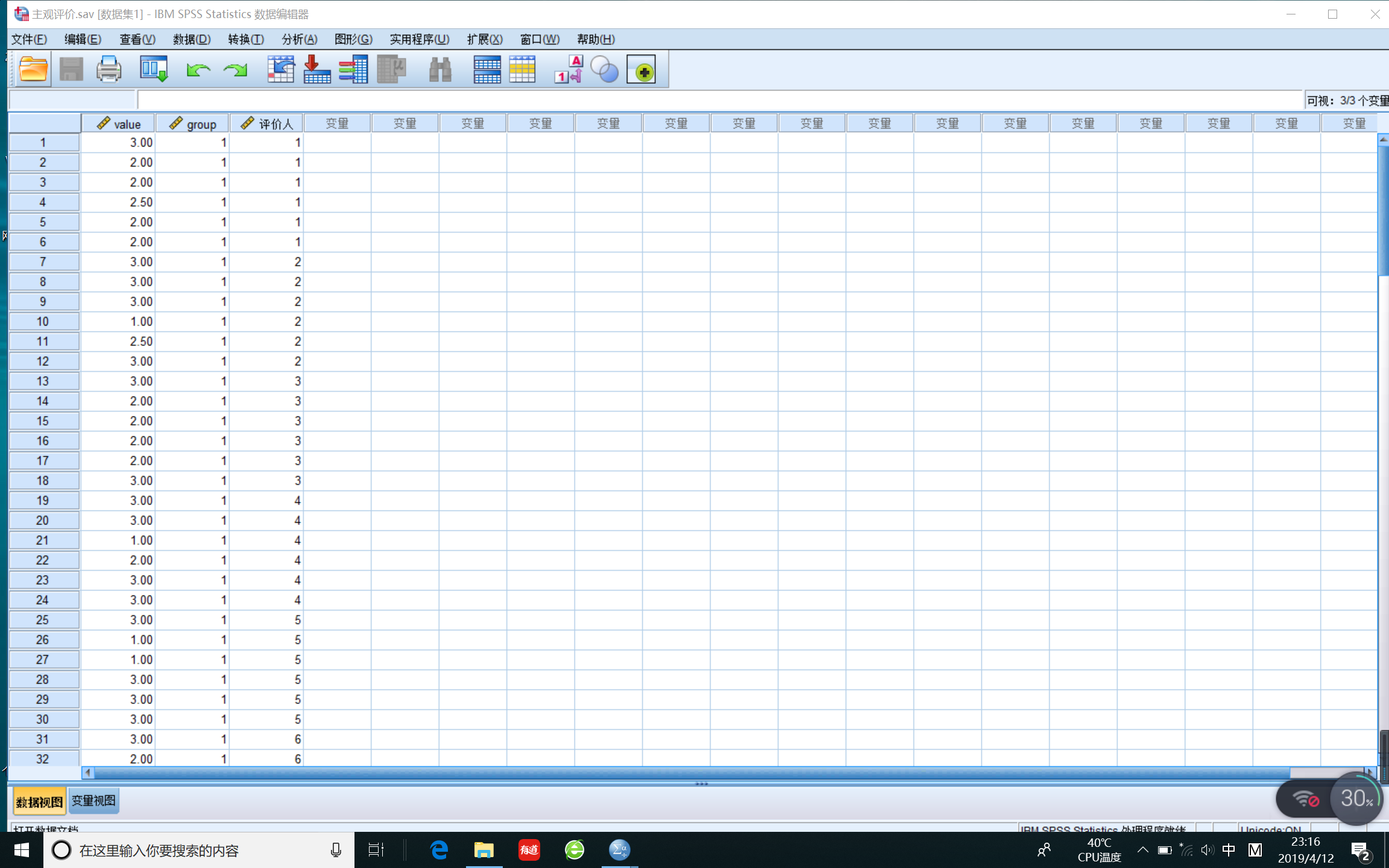

Supplement: S1 Fig — (TIF) [file pone.0227050.s001.tif]

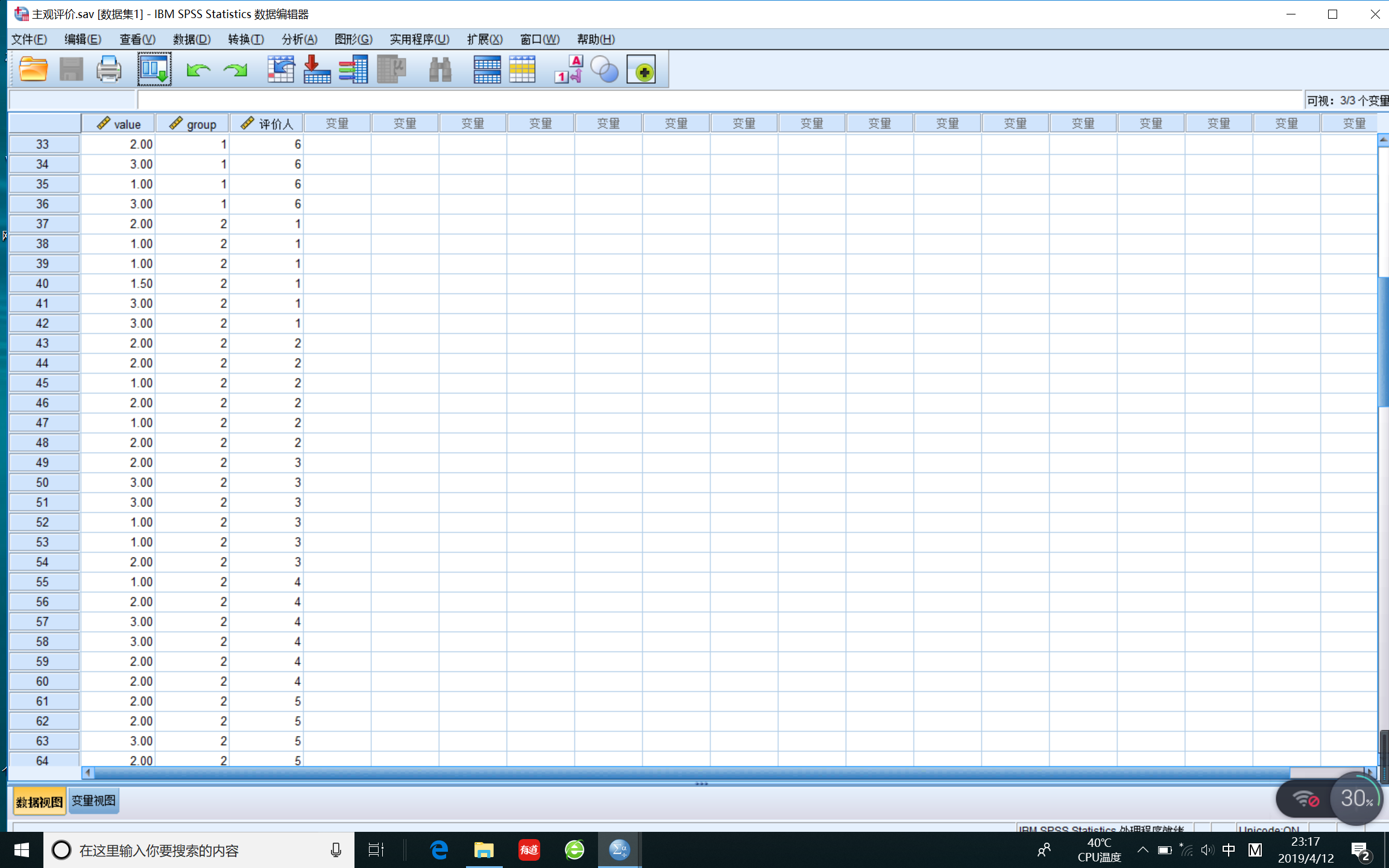

Supplement: S2 Fig — (TIF) [file pone.0227050.s002.tif]

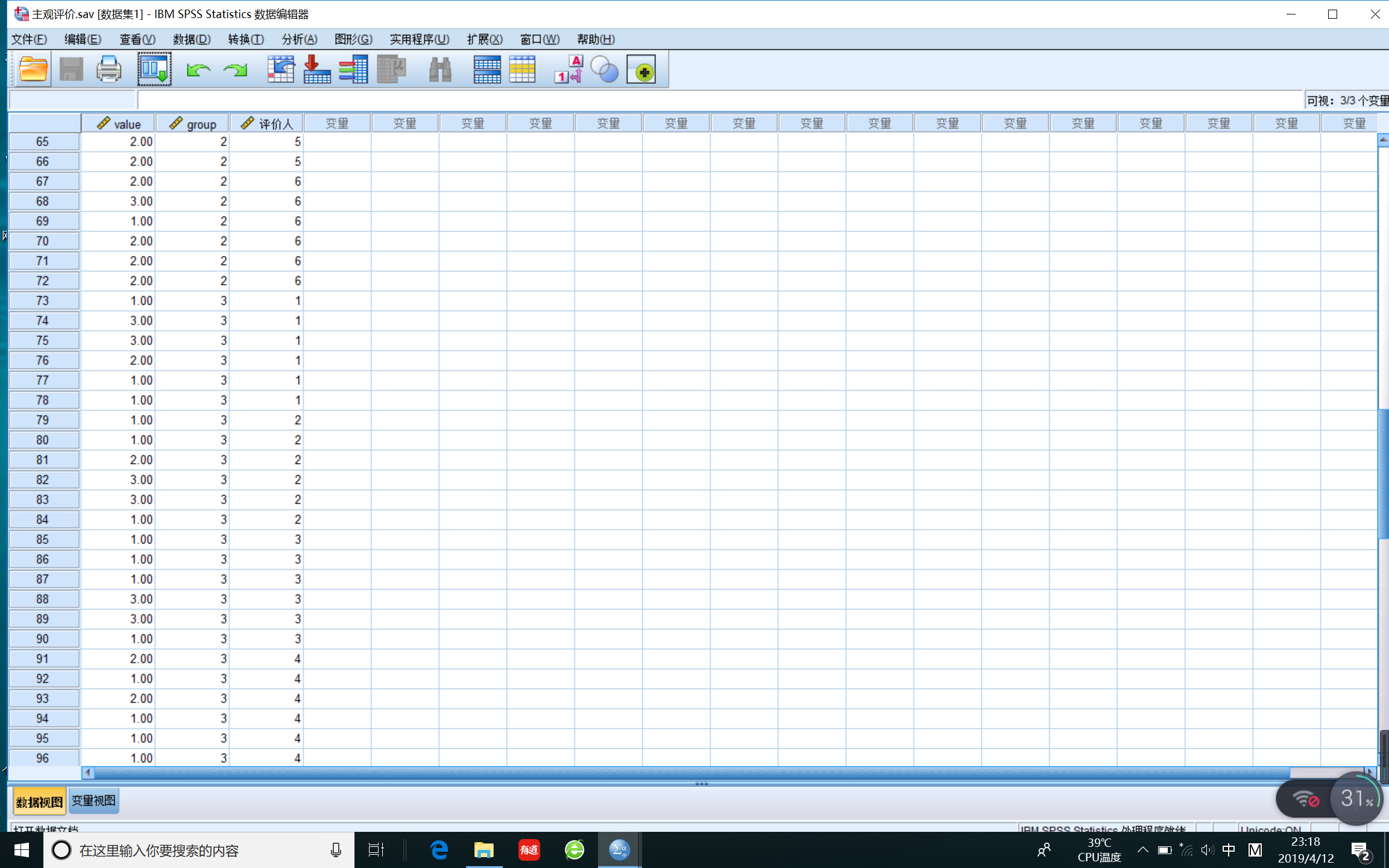

Supplement: S3 Fig — (TIF) [file pone.0227050.s003.tif]

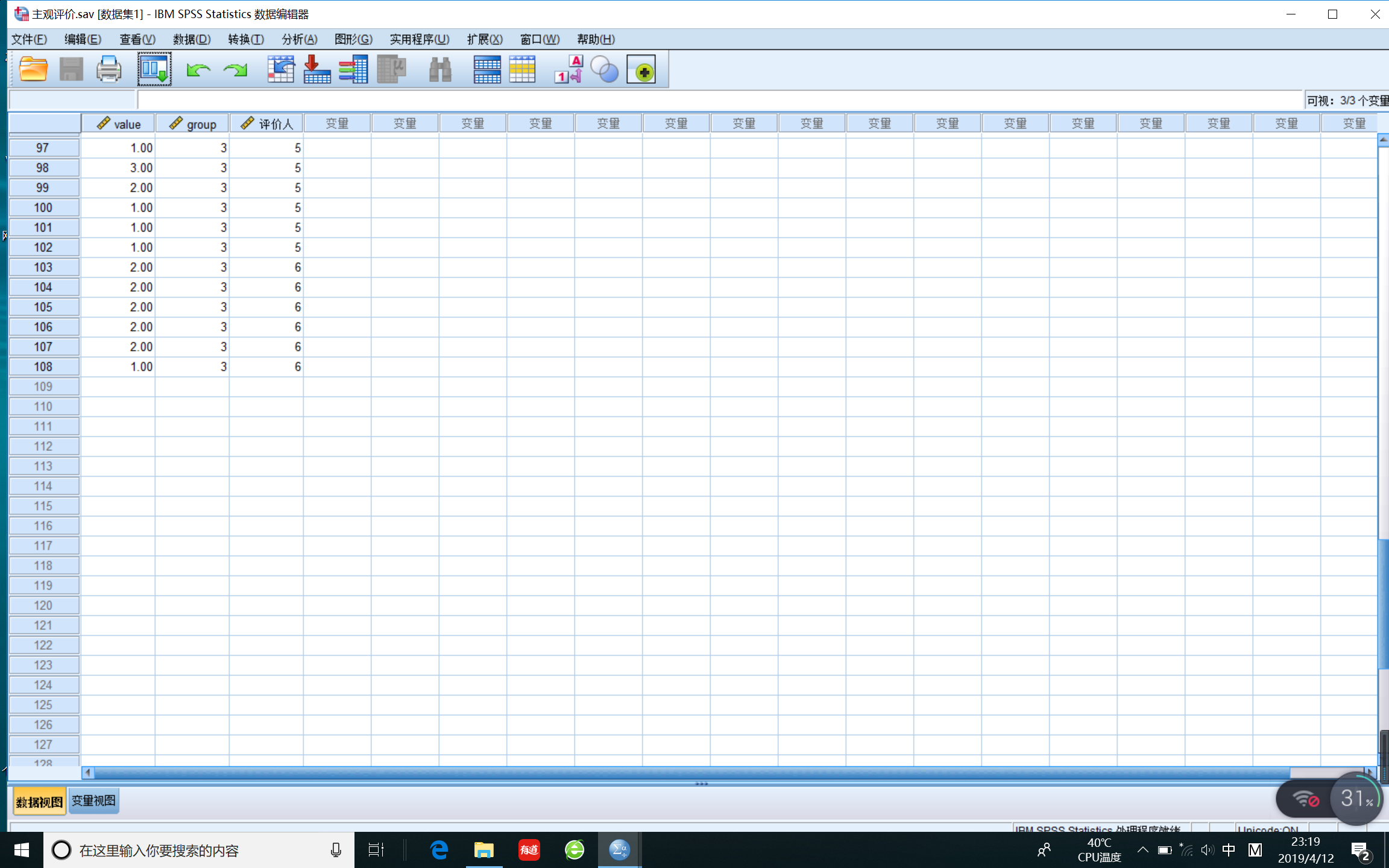

Supplement: S4 Fig — (TIF) [file pone.0227050.s004.tif]

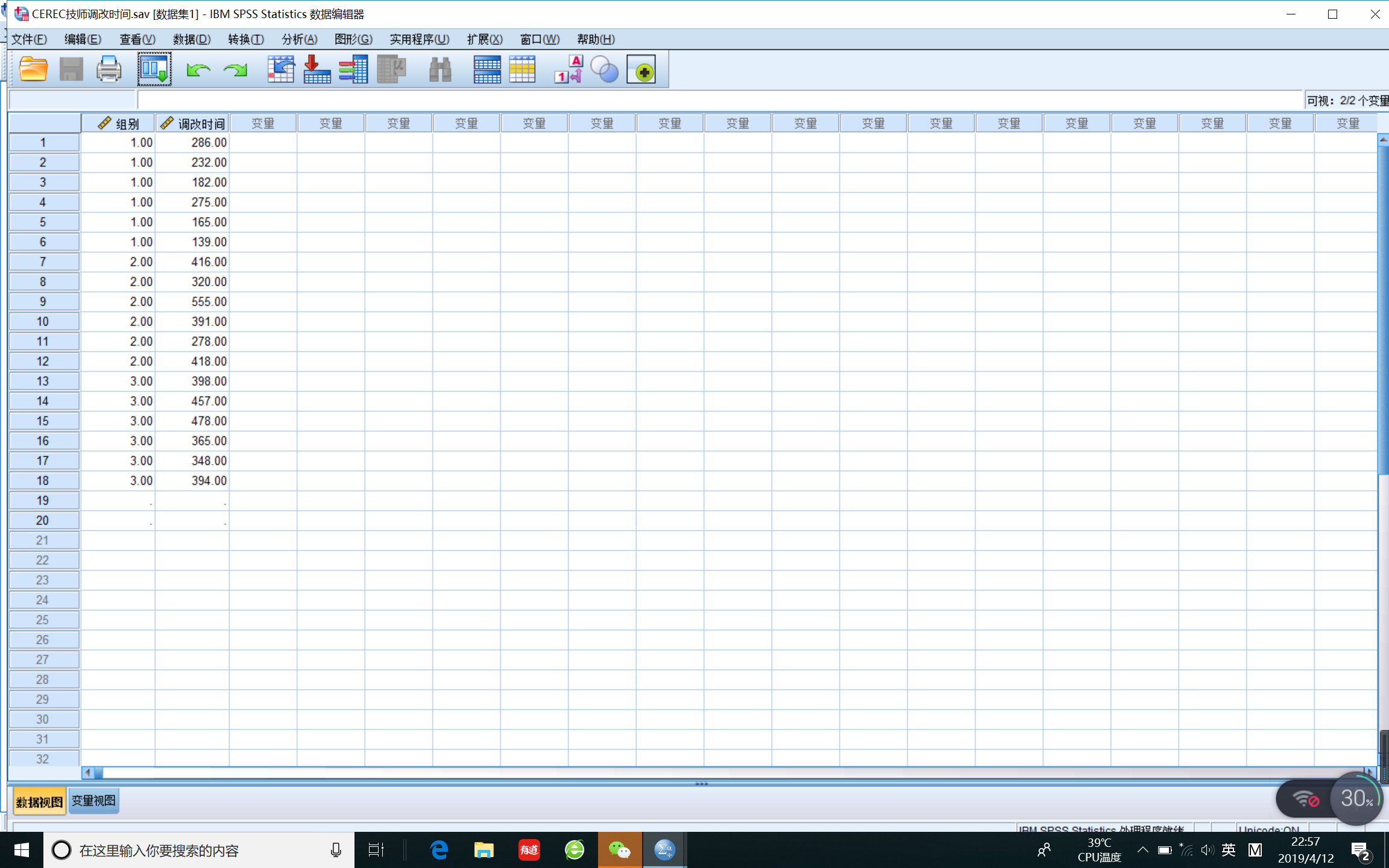

Supplement: S5 Fig — (TIF) [file pone.0227050.s005.tif]

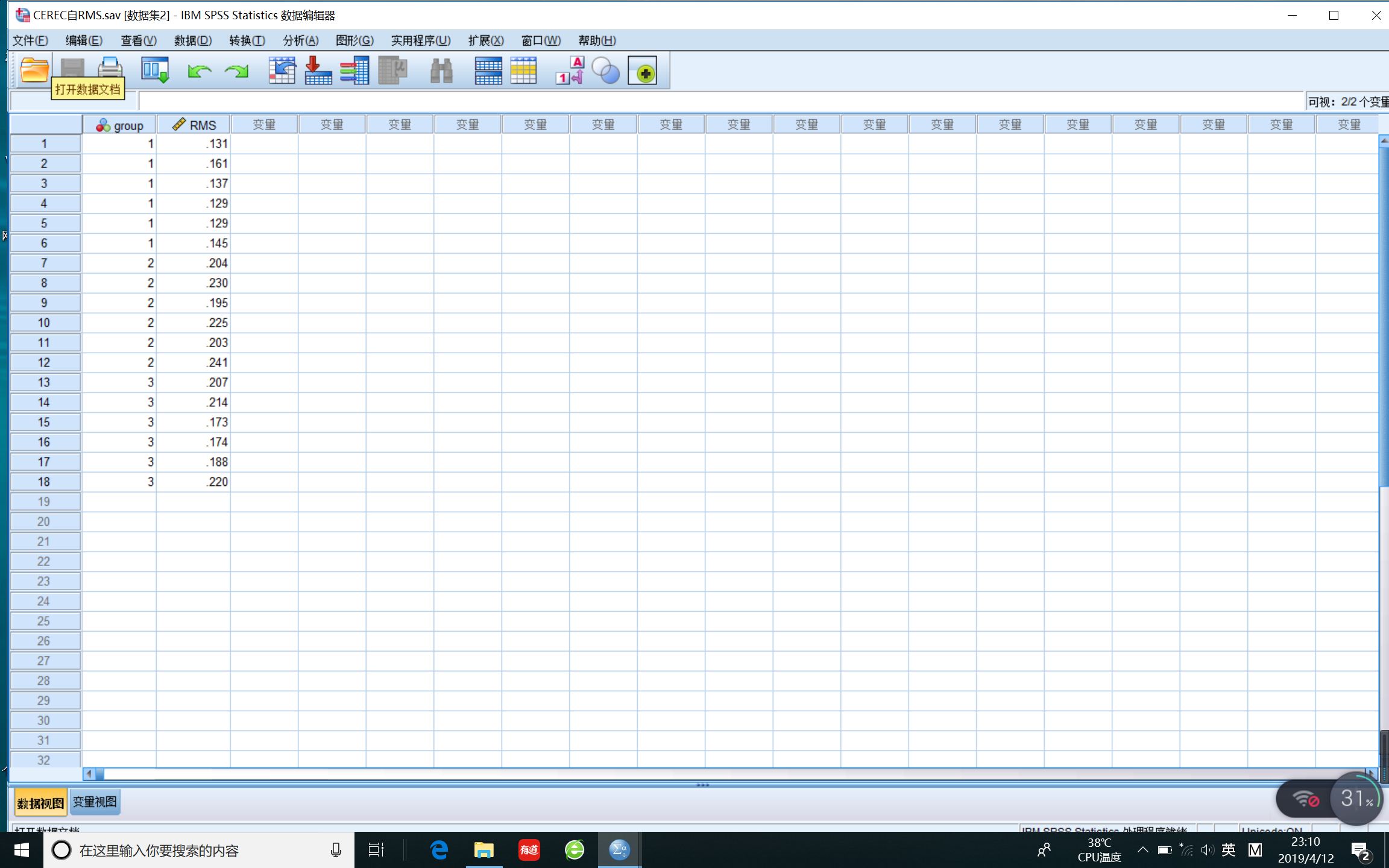

Supplement: S6 Fig — (TIF) [file pone.0227050.s006.tif]

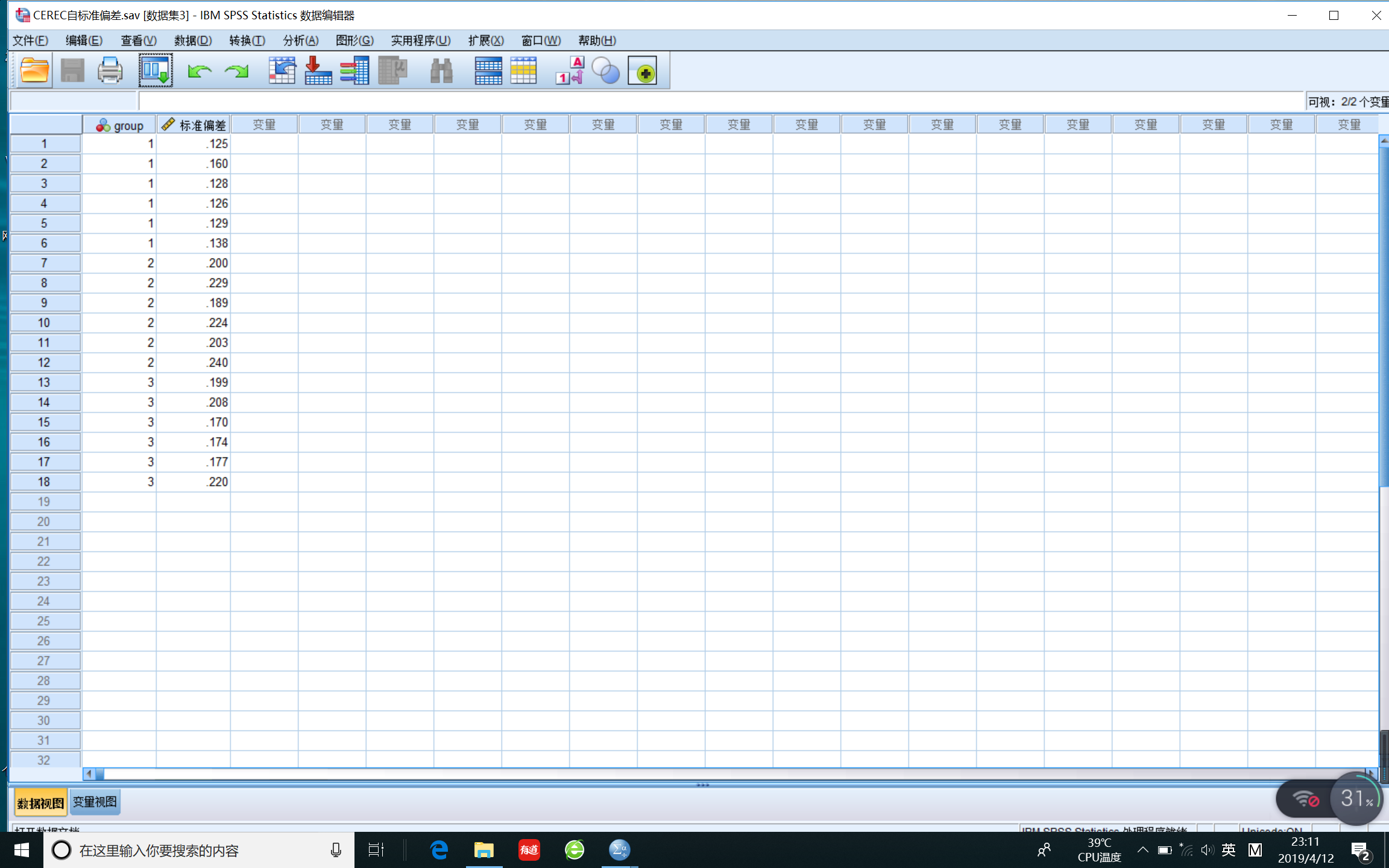

Supplement: S7 Fig — (TIF) [file pone.0227050.s007.tif]

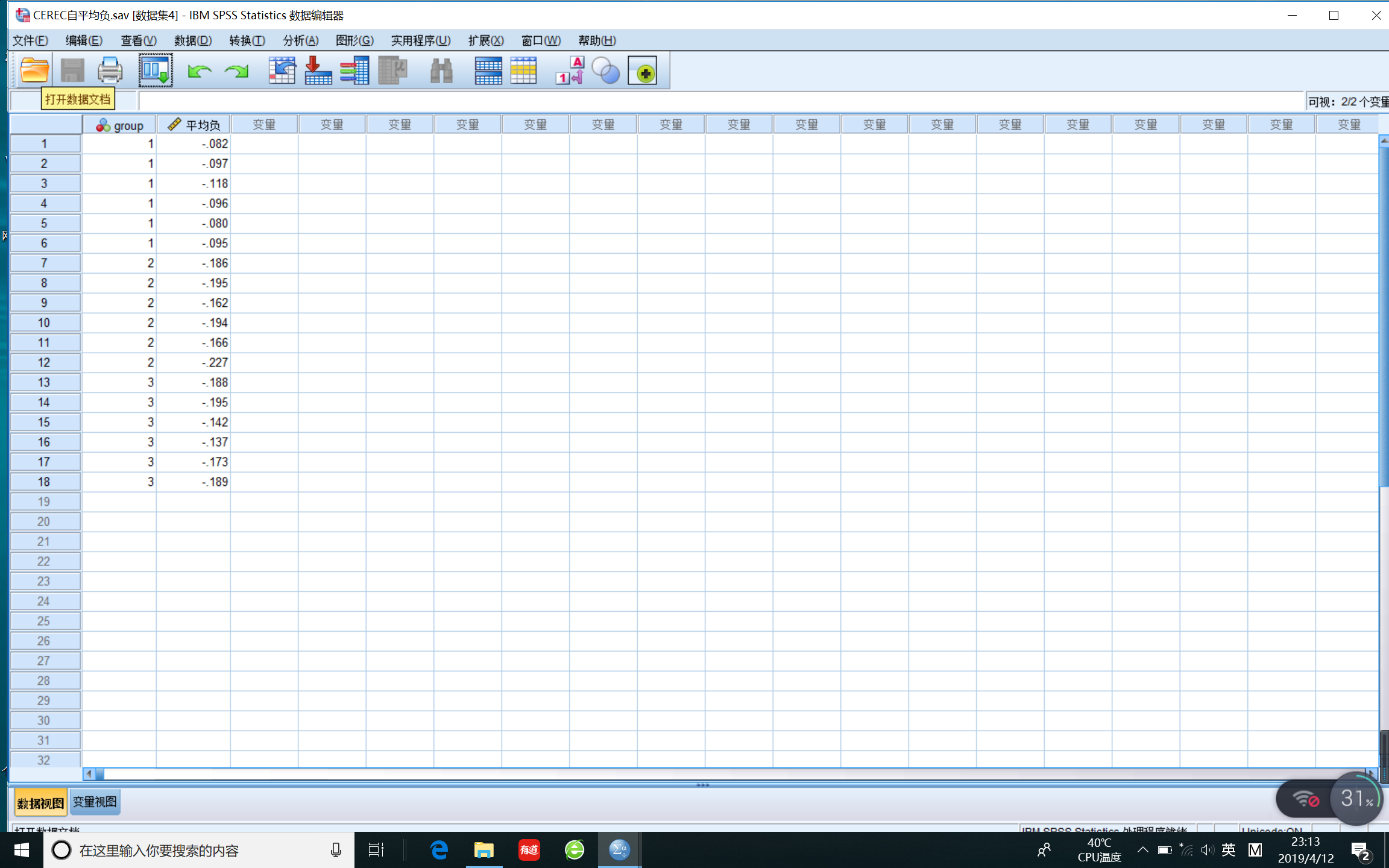

Supplement: S8 Fig — (TIF) [file pone.0227050.s008.tif]

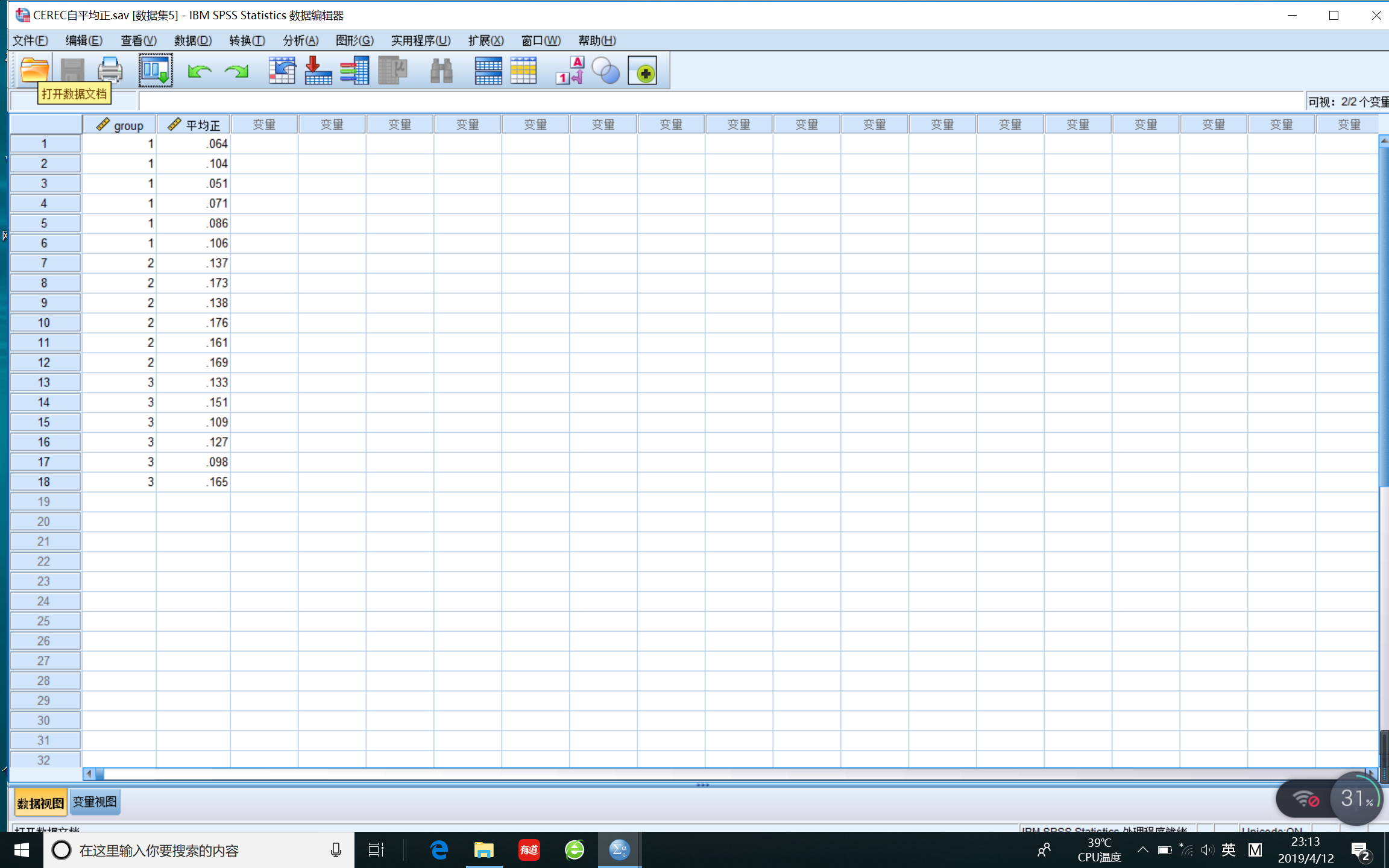

Supplement: S9 Fig — (TIF) [file pone.0227050.s009.tif]

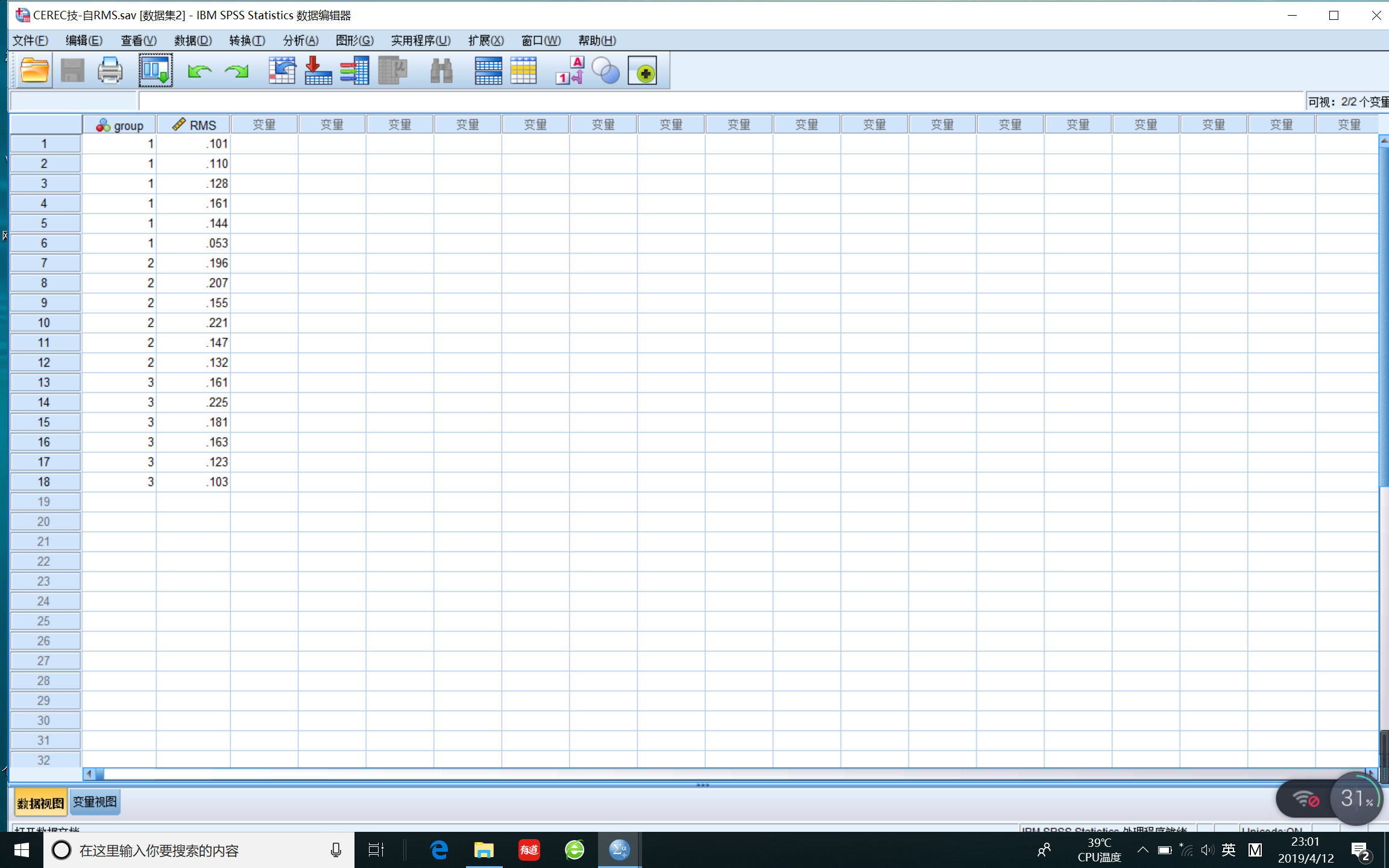

Supplement: S10 Fig — (TIF) [file pone.0227050.s010.tif]

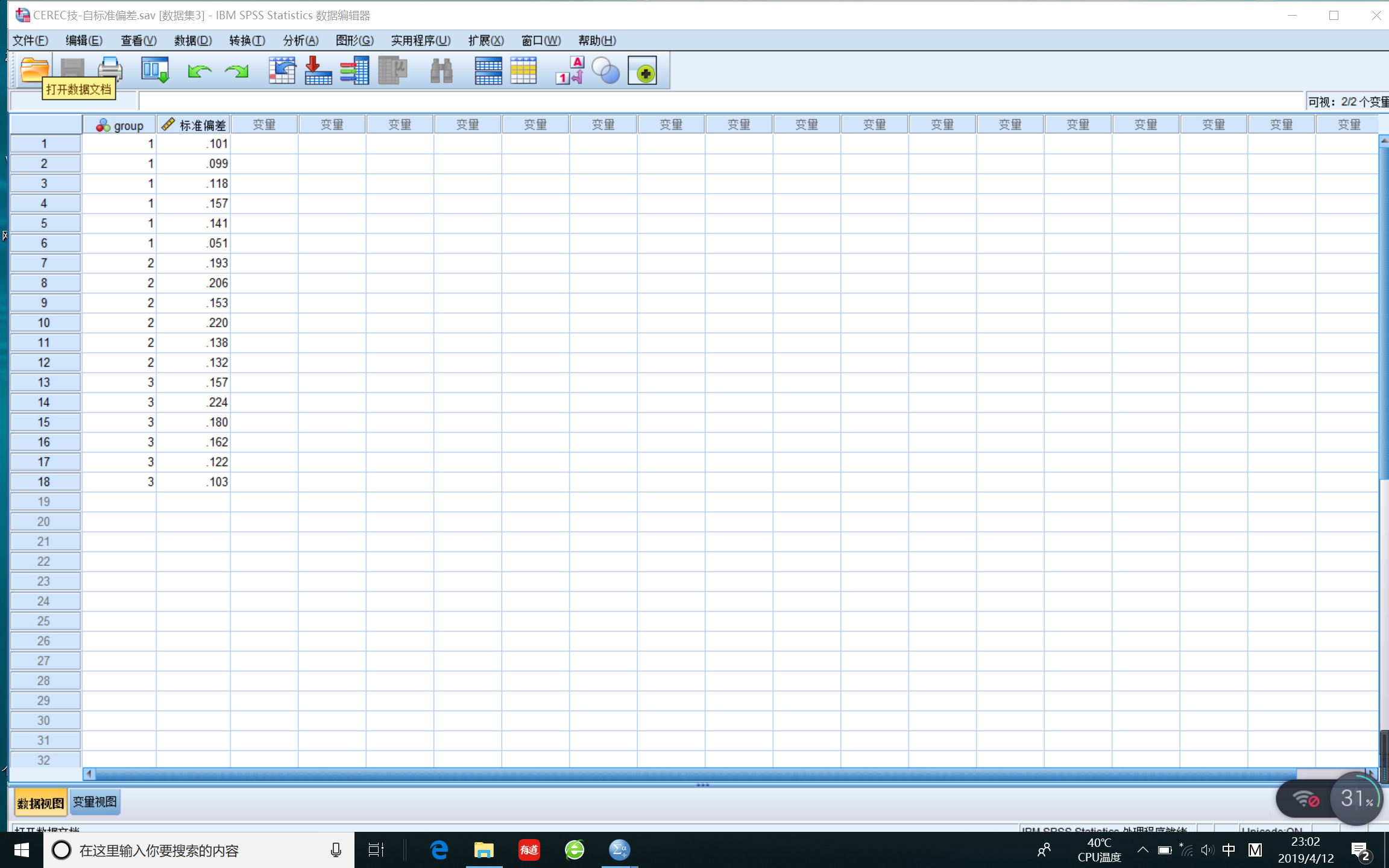

Supplement: S11 Fig — (TIF) [file pone.0227050.s011.tif]

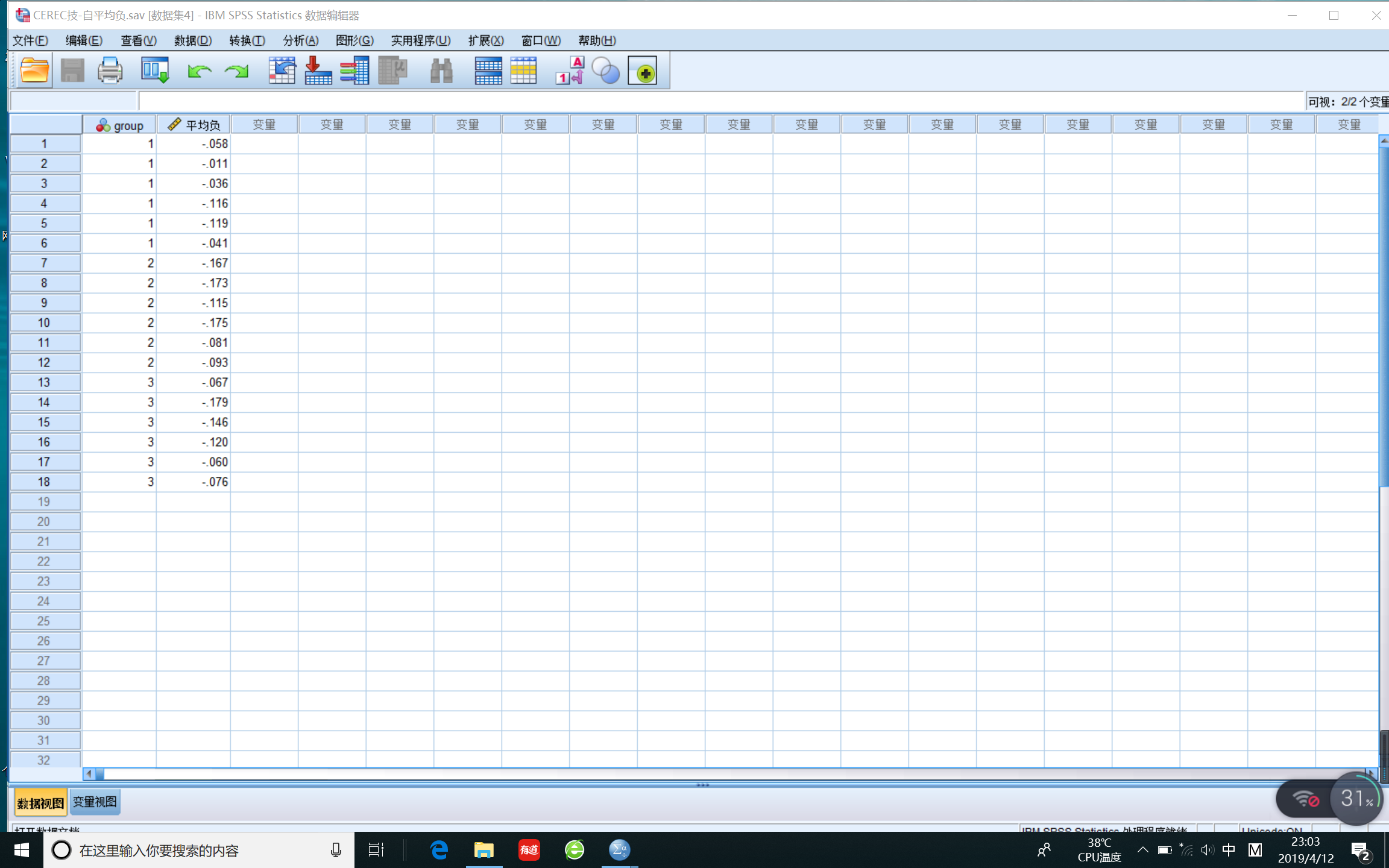

Supplement: S12 Fig — (TIF) [file pone.0227050.s012.tif]

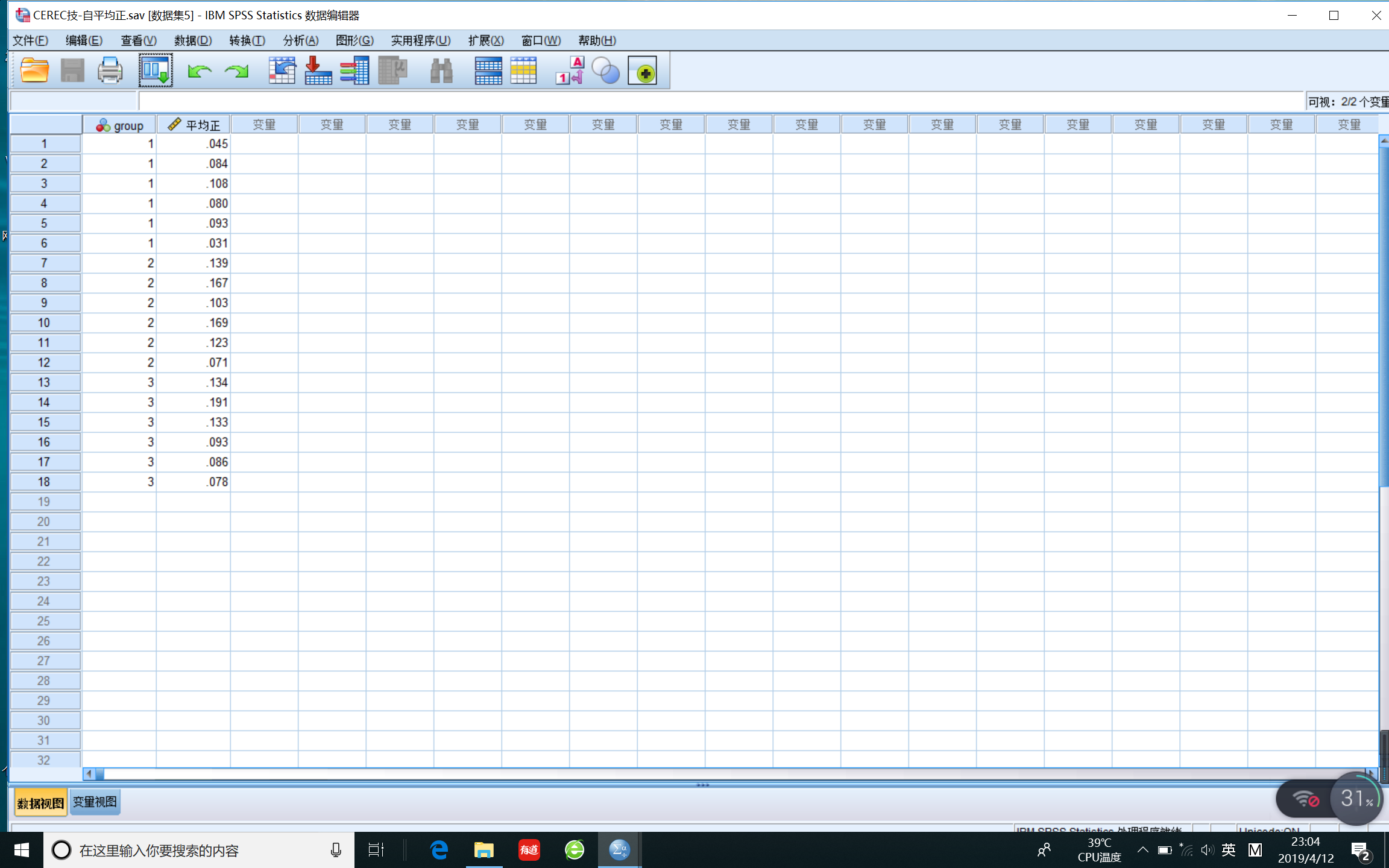

Supplement: S13 Fig — (TIF) [file pone.0227050.s013.tif]
